# Red blood cells as biomarkers and mediators in complications of diabetes mellitus: A review

**DOI:** 10.1097/MD.0000000000037265

**Published:** 2024-02-23

**Authors:** Emmanuel Ifeanyi Obeagu

**Affiliations:** a Department of Medical Laboratory Science, Kampala International University, Kampala, Uganda.

**Keywords:** biomarkers, diabetes, HbA1c, inflammation, red blood cells

## Abstract

Red blood cells (RBCs), traditionally recognized for their oxygen transport role, have garnered increasing attention for their significance as crucial contributors to the pathophysiology of diabetes mellitus. In this comprehensive review, we elucidate the multifaceted roles of RBCs as both biomarkers and mediators in diabetes mellitus. Amidst the intricate interplay of altered metabolic pathways and the diabetic milieu, RBCs manifest distinct alterations in their structure, function, and lifespan. The chronic exposure to hyperglycemia induces oxidative stress, leading to modifications in RBC physiology and membrane integrity. These modifications, including glycation of hemoglobin (HbA1c), establish RBCs as invaluable biomarkers for assessing glycemic control over extended periods. Moreover, RBCs serve as mediators in the progression of diabetic complications. Their involvement in vascular dysfunction, hemorheological changes, and inflammatory pathways contributes significantly to diabetic microangiopathy and associated complications. Exploring the therapeutic implications, this review addresses potential interventions targeting RBC abnormalities to ameliorate diabetic complications. In conclusion, comprehending the nuanced roles of RBCs as biomarkers and mediators in diabetes mellitus offers promising avenues for enhanced diagnostic precision, therapeutic interventions, and improved patient outcomes. This review consolidates the current understanding and emphasizes the imperative need for further research to harness the full potential of RBC-related insights in the realm of diabetes mellitus.

## 1. Introduction

Traditionally recognized for their primary role in oxygen transport, red blood cells (RBCs) have increasingly emerged as pivotal players in the pathophysiology and management of diabetes mellitus.^[1,2]^ The prevalence of diabetes mellitus has reached unprecedented levels worldwide, demanding a deeper comprehension of its intricate mechanisms and the identification of reliable biomarkers for early detection, prognosis, and effective management.^[3]^ Amidst this landscape, the unique properties of RBCs have garnered attention beyond their classic physiological function, extending into their potential roles as biomarkers and active mediators in the context of diabetes.

RBCs, the most abundant cellular components in the bloodstream, exhibit remarkable adaptability and responsiveness to the systemic alterations induced by hyperglycemia.^[4]^ Chronic exposure to elevated glucose levels triggers a cascade of events within these cells, leading to structural modifications, functional alterations, and changes in their biochemical composition.^[5]^ These modifications, ranging from increased oxidative stress to modifications in membrane properties and hemoglobin glycation, present opportunities for leveraging RBC characteristics as indicative markers reflecting the metabolic milieu over time.^[6]^ Beyond their utility as biomarkers, RBCs actively contribute to the intricate landscape of diabetic complications. Their involvement in the pathogenesis of microvascular complications, including retinopathy, neuropathy, and nephropathy, underscores their role as active mediators of vascular dysfunction and inflammation within the diabetic milieu.^[7]^

This paper aims to delve into the diverse facets of RBCs in the context of diabetes mellitus, elucidating their potential as both biomarkers and mediators. By exploring the alterations in RBC biology, their utility in monitoring glycemic control, and their contributions to diabetic complications, this comprehensive assessment seeks to highlight the multifaceted implications of RBCs in diabetes mellitus and underscores the avenues for future research and clinical applications.

## 2. RBCs in diabetes mellitus

RBCs play multifaceted roles in diabetes mellitus, extending beyond their conventional function of oxygen transportation.^[8]^ Certainly, in diabetic conditions, RBCs undergo various alterations in their structure, function, and lifespan due to the complex interplay of factors associated with hyperglycemia and its sequelae.^[9]^ Chronic exposure to high levels of glucose in the bloodstream leads to the non-enzymatic glycated hemoglobin (HbA1c) molecules within RBCs. This process results in the formation of HbA1c, which reflects the average blood glucose levels over a prolonged period. Increased levels of HbA1c are indicative of poor glycemic control in diabetes.^[10,11]^ Hyperglycemia can induce modifications in the RBC membrane, affecting its composition, fluidity, and stability. These alterations often lead to changes in membrane permeability, impairments in ion transport mechanisms, and alterations in membrane-bound enzymes, affecting overall RBC functionality.^[12]^ HbA1c affects its ability to bind and release oxygen efficiently. This alteration can disrupt the normal oxygen-carrying capacity of RBCs, impacting oxygen delivery to tissues and organs.^[13]^ RBCs in diabetic conditions often exhibit reduced flexibility or deformability, hindering their ability to navigate through narrow capillaries and microvessels. This reduced deformability contributes to impaired microcirculation and tissue perfusion.^[14]^ Chronic exposure to hyperglycemia and oxidative stress can accelerate the aging process of RBCs, leading to premature cell death or hemolysis. Consequently, RBCs may have a shortened lifespan compared to those in non-diabetic individuals.^[15]^ RBCs in diabetes are more susceptible to oxidative damage due to elevated levels of reactive oxygen species (ROS) generated as a consequence of hyperglycemia. This increased oxidative stress can further contribute to RBC dysfunction and premature aging.^[16]^ These alterations collectively contribute to the overall impairment of RBC structure, function, and longevity in diabetes mellitus.^[17]^ The changes in RBC properties not only impact their primary function of oxygen transport but also play a significant role in the development and progression of diabetic complications, particularly those related to microvascular dysfunction. Understanding these alterations in RBC biology is crucial for developing targeted interventions aimed at preserving RBC function and ameliorating the impact of diabetic complications on microcirculation and tissue health.

## 3. Contribution to diabetic complications

RBC abnormalities, such as altered deformability and increased adhesiveness, can contribute to impaired retinal microcirculation.^[18]^ Reduced blood flow and microvascular changes in the retina are key factors in the development of diabetic retinopathy, a leading cause of vision impairment and blindness.^[19,20]^ Dysfunctional RBCs may contribute to renal microvascular damage and impairments in kidney function. Altered blood flow and reduced oxygen delivery to the kidneys are associated with the development of diabetic nephropathy, a major cause of end-stage renal disease.^[21]^ Impaired microcirculation due to abnormal RBC properties contributes to nerve damage in diabetic neuropathy. Reduced oxygen supply and microvascular dysfunction play a role in peripheral nerve damage, resulting in sensory and motor neuropathies.^[22]^ Diabetes is a major risk factor for cardiovascular complications. Altered RBC properties, such as reduced deformability and increased oxidative stress, contribute to impaired blood flow and endothelial dysfunction, thereby exacerbating the risk of atherosclerosis, coronary artery disease, and stroke.^[23,24]^ Changes in RBC functionality, including impaired oxygen delivery due to decreased deformability and altered hemoglobin function, contribute to tissue hypoxia. Prolonged tissue hypoxia can lead to cellular damage and contribute to the pathogenesis of diabetic complications in various organs.^[25]^ Dysfunctional RBCs can contribute to the systemic inflammatory milieu seen in diabetes. Interaction between RBCs and endothelial cells, as well as the release of inflammatory mediators from RBCs, can contribute to chronic inflammation and exacerbate tissue damage.^[26]^ Abnormal RBCs in diabetes generate and are exposed to increased oxidative stress, contributing to systemic oxidative damage. This oxidative stress plays a role in the development and progression of diabetic complications in multiple organ systems.^[27]^ Dysfunctional RBCs may interact with endothelial cells, affecting nitric oxide (NO) bioavailability and endothelial function. This interaction contributes to endothelial dysfunction, impairing vascular health and promoting the development of diabetic vascular complications.^[28]^ Understanding the role of RBC abnormalities in diabetic complications emphasizes the importance of preserving RBC function and addressing microvascular alterations in diabetes management. Strategies aimed at improving RBC health, mitigating oxidative stress, and preserving microvascular function are crucial in reducing the burden of diabetic complications.

## 4. Role of hyperglycemia in inducing oxidative stress and impacting RBCs

Hyperglycemia, a hallmark of diabetes, plays a pivotal role in inducing oxidative stress and significantly impacting RBCs. The chronic exposure of RBCs to elevated glucose levels leads to various biochemical and physiological changes that contribute to oxidative stress and impair RBC function.^[29]^ Elevated blood glucose levels promote the non-enzymatic HbA1c within RBCs. This process leads to the formation of HbA1c, which serves as a marker for long-term glucose control. Increased HbA1c levels are associated with higher risks of diabetic complications.^[30]^ Hyperglycemia induces the production of ROS within RBCs through various pathways, such as the polyol pathway, advanced glycation end products (AGEs) formation, and increased mitochondrial oxidative stress.^[31]^ Prolonged exposure to high glucose impairs the antioxidant defense mechanisms in RBCs, leading to a reduction in antioxidant enzymes (e.g., superoxide dismutase, catalase) and decreased levels of intracellular antioxidants (e.g., glutathione). This imbalance between ROS production and antioxidant defense exacerbates oxidative stress.^[32]^ Hyperglycemia contributes to modifications in the RBC membrane, such as altered lipid composition and increased protein glycation. These changes lead to decreased membrane fluidity and increased rigidity, impairing RBC deformability and their ability to pass through microvasculature.^[33]^ Glycated hemoglobin exhibits altered oxygen affinity, affecting the normal release and delivery of oxygen to tissues. This disruption in oxygen transport can contribute to tissue hypoxia, further exacerbating oxidative stress and cellular damage.^[34]^ The increased oxidative stress and structural alterations induced by hyperglycemia can shorten the lifespan of RBCs. Premature aging or hemolysis of RBCs occurs due to oxidative damage, leading to a decreased RBC lifespan compared to non-diabetic conditions.^[35]^ The impact of hyperglycemia-induced oxidative stress on RBCs is implicated in the development and progression of diabetic complications, particularly microvascular complications like retinopathy, nephropathy, and neuropathy.^[36]^ Understanding the mechanisms by which hyperglycemia induces oxidative stress in RBCs is crucial in comprehending the pathophysiology of diabetic complications. Strategies aimed at mitigating oxidative stress in RBCs, preserving their functionality, and maintaining a redox balance may hold therapeutic potential in managing diabetes and reducing the burden of associated complications.

## 5. HbA1c as a diagnostic marker for glycemic control

HbA1c, specifically the measurement of HbA1c, serves as a valuable diagnostic marker and an essential tool for assessing long-term glycemic control in individuals with diabetes mellitus.^[37]^ HbA1c provides an indication of an individual average blood glucose levels over the past 2 to 3 months, representing a longer-term measure compared to routine blood glucose monitoring, which captures only point-in-time measurements. HbA1c is formed by the non-enzymatic attachment of glucose to hemoglobin molecules in RBCs. As RBCs circulate in the bloodstream, HbA1c is generated proportionally to the level of glucose in the blood, reflecting the average exposure of hemoglobin to glucose during the lifespan of RBCs (around 120 days).^[38]^ Measurement of HbA1c levels is integral in assessing the efficacy of diabetes management and treatment strategies. It aids clinicians in determining whether a patient blood glucose levels have been adequately controlled over a more extended period.^[39]^ Guidelines often set target HbA1c levels to guide diabetes management. Lowering HbA1c levels within recommended targets reduces the risk of diabetic complications.^[40]^ HbA1c monitoring encourages patient compliance with treatment plans and allows healthcare providers to make necessary adjustments to medications, lifestyle interventions, and management strategies based on the patient glycemic control status.^[41]^ Factors like variations in RBC lifespan, certain medical conditions affecting hemoglobin, and conditions that impact erythrocyte turnover can affect HbA1c measurements, leading to some variability among individuals.^[42]^ HbA1c measurements might not accurately reflect glycemic control in certain populations, such as individuals with conditions affecting erythrocyte turnover, anemia, or specific hemoglobin variants (hemoglobinopathies).^[43]^ Specific HbA1c thresholds are used for diagnosing diabetes and prediabetes according to established guidelines. For instance, an HbA1c level of 6.5% or higher is typically indicative of diabetes, while levels between 5.7% and 6.4% may indicate prediabetes.^[44]^ HbA1c measurement serves as a valuable tool in clinical practice, offering insights into long-term glycemic control and aiding in the assessment of diabetes management effectiveness.^[45]^ While it provides a reliable marker for average blood glucose levels over time, healthcare providers must consider individual factors and limitations when interpreting HbA1c measurements for optimal diabetes care.

## 6. Influence of diabetic microvascular complications on RBC physiology

Diabetic microvascular complications exert significant influence on RBC physiology due to the intricate interplay between altered microcirculation, oxidative stress, and biochemical changes associated with diabetes.^[46]^ Microvascular complications such as diabetic retinopathy, nephropathy, and neuropathy often lead to reduced blood flow and compromised microcirculation in various tissues and organs.^[47]^ Decreased oxygen supply to tissues due to impaired microcirculation can affect RBC deformability. RBCs become less flexible, hindering their ability to traverse through microvessels and deliver oxygen efficiently to tissues.^[48]^ Microvascular complications contribute to an environment of increased oxidative stress. RBCs circulating through damaged microvessels are exposed to heightened levels of ROS and oxidative damage, affecting their structural integrity and function.^[49,50]^ Chronic exposure to the oxidative milieu in damaged microvasculature can compromise the antioxidant defense mechanisms within RBCs, further exacerbating oxidative stress.^[51]^ Oxidative stress and exposure to an abnormal microenvironment can accelerate the aging process of RBCs. This premature aging might result in a shortened lifespan for RBCs compared to those in non-diabetic conditions.^[52]^ Prolonged exposure of RBCs to oxidative stress and altered microcirculation can affect hemoglobin functionality. HbA1c and oxidative modifications can impair oxygen-binding capacity and contribute to dysfunctional oxygen transport.^[53,54]^ Damaged microvasculature and compromised tissue perfusion associated with microvascular complications contribute to the release of inflammatory mediators. RBCs, when exposed to this inflammatory milieu, might contribute to the systemic inflammatory response observed in diabetic microangiopathy.^[55,56]^ These alterations in RBC physiology as a consequence of microvascular complications impact oxygen delivery, tissue perfusion, and cellular health, potentially exacerbating tissue damage and contributing to the progression of diabetic complications in affected organs.^[57]^ Understanding the influence of microvascular complications on RBC physiology is crucial in comprehending the systemic impact of diabetes on vascular health and tissue integrity. It underscores the importance of strategies aimed at preserving RBC functionality and mitigating microvascular damage to reduce the burden of diabetic complications.

## 7. RBCs as biomarkers in diabetes mellitus

RBCs and associated parameters serve as informative biomarkers in the context of diabetes mellitus, offering insights into various aspects of the disease progression and management.^[58]^ HbA1c is a key biomarker used to assess average blood glucose levels over a span of 2 to 3 months. Elevated HbA1c levels indicate poor glycemic control and are associated with an increased risk of diabetic complications. It remains a cornerstone for diabetes management, aiding in treatment decisions and assessing the effectiveness of interventions.^[59]^ Changes in RBC count, size (mean corpuscular volume), and indices (mean corpuscular hemoglobin, mean corpuscular hemoglobin concentration) can provide insights into underlying conditions or complications associated with diabetes, such as anemia or other hematological abnormalities. RBCs are susceptible to oxidative stress in diabetes, leading to increased production of ROS. Assessing markers of oxidative stress within RBCs, such as lipid peroxidation products or antioxidant enzyme activities, can offer insights into oxidative damage associated with the disease.^[60]^ Changes in RBC membrane properties, including alterations in deformability and stability, are observed in diabetes. Assessing these changes may provide information about microvascular complications and altered hemodynamics.^[61]^ Certain hemoglobin variants or conditions affecting RBC turnover can influence HbA1c measurements or RBC parameters, warranting consideration in the interpretation of diagnostic results, particularly in populations with specific genetic traits or blood disorders.^[62]^

## 8. Advanced biomarkers and technologies

Ongoing research explores novel RBC-related biomarkers beyond traditional parameters, aiming to identify more specific indicators of diabetes progression, vascular health, or oxidative stress within RBCs.^[63]^ High-resolution imaging techniques, proteomic analysis, and metabolomic profiling offer opportunities for a deeper understanding of RBC-related changes and the development of more precise diagnostic tools for diabetes management.^[64]^ RBC-related biomarkers serve as valuable tools in diagnosing and monitoring diabetes mellitus, assessing glycemic control, evaluating complications, and guiding treatment strategies.^[65]^ Their assessment aids in personalized management approaches, especially when considering individual variations and factors that might influence RBC parameters, ensuring a more comprehensive understanding of the disease and its impact on hematological health. RBC-related biomarkers play a multifaceted role in diabetes mellitus, providing valuable information regarding glycemic control, hematological alterations, oxidative stress, and potential complications associated with the disease. Continuous research into RBC-specific markers offers prospects for improved diagnostic accuracy and more targeted therapeutic interventions in diabetes management.

## 9. RBCs as mediators of diabetic complications

RBCs play a significant role as mediators in the development and progression of diabetic complications. The alterations and interactions involving RBCs contribute to various pathological processes, particularly in the context of microvascular complications associated with diabetes.^[57]^ Altered properties of RBCs, such as reduced deformability and increased adhesiveness, contribute to microvascular dysfunction in diabetes. These changes hinder RBCs’ ability to flow through small vessels, compromising tissue perfusion.^[66]^ RBCs are exposed to increased oxidative stress due to prolonged hyperglycemia in diabetes. This leads to the generation of ROS within RBCs, contributing to systemic oxidative stress.^[67]^ RBCs, when exposed to an oxidative environment, release inflammatory mediators and cytokines, contributing to chronic inflammation and endothelial dysfunction, key factors in the development of diabetic complications.^[67]^ Abnormal RBC-endothelial cell interactions impair vascular endothelial function. RBCs influence NO bioavailability and endothelial cell integrity, impacting vascular tone and endothelial health.^[68]^ Altered RBC properties, including impaired deformability, contribute to decreased oxygen delivery to tissues. Tissue hypoxia is a key factor in the development of diabetic complications, including retinopathy, nephropathy, and neuropathy.^[67]^ Prolonged tissue hypoxia due to impaired RBC function can lead to cellular damage and contribute to the progression of diabetic complications in various organs.^[69]^ RBCs, when exposed to the diabetic milieu, undergo changes that make them more prone to interaction with endothelial cells and immune cells. These interactions contribute to systemic inflammation and amplify the inflammatory response associated with diabetes.^[70]^ The involvement of RBCs as mediators in microvascular dysfunction, oxidative stress, and inflammation contributes significantly to the pathogenesis and progression of diabetic complications.^[71]^

Strategies aimed at preserving RBC function, improving deformability, and mitigating oxidative stress within RBCs may hold promise in managing and preventing diabetic microvascular complications.^[72]^ Understanding the role of RBCs as mediators in diabetic complications is crucial for developing targeted interventions aimed at preserving RBC functionality and ameliorating the impact of diabetes on microvascular health. Strategies focusing on reducing oxidative stress, improving RBC deformability, and addressing inflammatory responses may offer potential therapeutic avenues in managing diabetic complications and improving patient outcomes.

## 10. Therapeutic implications and future perspectives

Developing therapies that specifically target and reduce oxidative stress within RBCs could potentially mitigate cellular damage and complications associated with diabetes. Antioxidant interventions might help restore RBC functionality and reduce the burden of oxidative damage.^[73]^ Interventions aimed at improving RBC deformability may help restore microvascular function and enhance oxygen delivery to tissues, potentially alleviating tissue hypoxia associated with diabetic complications.^[74]^ Research into novel drugs targeting RBC membrane alterations, oxidative stress pathways, or inflammation specifically within RBCs could lead to innovative therapeutic options aimed at preserving RBC health and function in diabetes. Advancements in nanotechnology might offer opportunities for targeted drug delivery to RBCs, allowing for more efficient and specific therapeutic interventions.^[75]^ Tailoring treatments based on individual variations in RBC-related markers could enable personalized management approaches, optimizing therapies according to patients’ specific needs and risks.^[76]^

## 11. Biomarker development and monitoring

Continued research to identify and validate new RBC-related biomarkers beyond HbA1c could enhance early detection, prognosis, and monitoring of diabetic complications, providing more comprehensive insights into disease progression. Development of point-of-care devices or assays for rapid and accurate assessment of RBC-related biomarkers could facilitate real-time monitoring and improve clinical decision-making. Integrating knowledge from multiple disciplines, including hematology, biochemistry, and nanotechnology, may lead to more comprehensive approaches targeting RBCs in diabetes management.^[77]^ Considering combination therapies that address multiple aspects of RBC alterations (e.g., oxidative stress, membrane integrity) simultaneously may yield synergistic effects in managing diabetic complications. Further research into the molecular mechanisms underlying RBC alterations in diabetes, exploring potential therapeutic targets, and conducting rigorous clinical trials are essential to translate findings into clinical practice. Investigating the long-term effects of targeted interventions on RBC health and diabetic complications will be crucial for assessing their efficacy and safety. Leveraging the insights into RBC-related alterations in diabetes offers potential therapeutic avenues and future perspectives for developing targeted interventions. Innovative strategies focusing on oxidative stress reduction, improving RBC functionality, personalized medicine, and integrative approaches hold promise in enhancing diabetes management and reducing the burden of associated complications. Continued research and advancements in this field will likely pave the way for more effective therapies and improved outcomes for individuals living with diabetes.

## 12. Hemolysis with RBC hyperaggregability in diabetes

Hemolysis refers to the breakdown or destruction of RBCs, which can occur due to various factors.^[78]^ In diabetes, particularly in uncontrolled or poorly managed cases, several mechanisms contribute to alterations in RBC function, leading to increased susceptibility to hemolysis and hyperaggregability.^[79]^ Elevated blood glucose levels in diabetes can lead to increased osmotic stress on RBCs. When blood sugar levels are high, excess glucose molecules enter the RBCs, creating an osmotic gradient that draws water into the cells. This influx of water causes swelling of RBCs, making them more fragile and susceptible to premature destruction (hemolysis) when passing through narrow capillaries.^[80]^ Chronic exposure to high glucose levels leads to the non-enzymatic glycation of proteins within RBCs. This process involves the binding of glucose molecules to proteins, altering their structure and function. Glycation affects various RBC membrane proteins and can impair their flexibility and deformability, making RBCs more prone to premature destruction and decreasing their ability to navigate through microvasculature.^[81]^ Diabetes often leads to increased oxidative stress due to the generation of excessive ROS. These ROS can damage RBC membranes, proteins, and lipids, further impairing RBC function and stability. Oxidative stress contributes to the fragility of RBCs, making them more susceptible to hemolysis.^[82]^ Hyperaggregability refers to the increased tendency of RBCs to form aggregates or clumps. In diabetes, alterations in the RBC membrane composition and function, such as changes in lipid content and membrane proteins due to glycation and oxidative stress, can lead to increased RBC aggregation. These aggregates can impede blood flow through the microvasculature, contributing to tissue hypoxia and complications associated with diabetes.^[83]^ Managing diabetes through proper blood sugar control, lifestyle modifications, and medications as prescribed by healthcare providers can help mitigate these effects. Additionally, antioxidants and therapies targeting oxidative stress may have a beneficial impact on reducing RBC damage and subsequent hemolysis.^[84]^ Regular monitoring and management of diabetes-related complications, including those affecting RBC function, are crucial to prevent or minimize the adverse effects on various organs and tissues caused by hemolysis and hyperaggregability in diabetes. Always consult healthcare professionals for personalized guidance and treatment plans related to diabetes management.

## 13. Hemorheological changes on micro-angiopathic complications in diabetes

Hemorheology refers to the study of blood flow and its related properties, including viscosity, deformability of blood cells (such as RBCs), and the behavior of blood under various conditions.^[85]^ In diabetes, micro-angiopathic complications often arise due to alterations in hemorheological parameters, impacting blood flow and microcirculation in small blood vessels.^[86]^ Diabetes can lead to increased blood viscosity, primarily due to higher levels of plasma proteins, such as fibrinogen and globulins, as well as elevated levels of lipids and hyperglycemia-induced changes in blood components. Increased viscosity can impede blood flow through the microvasculature, leading to reduced perfusion and oxygen delivery to tissues.^[87]^ Changes in the structure and function of RBCs occur in diabetes, affecting their deformability—the ability to change shape and squeeze through narrow capillaries. Glycation, oxidative stress, and alterations in membrane composition compromise RBC flexibility, reducing their ability to navigate through microvessels. This impaired deformability contributes to microcirculatory dysfunction and tissue hypoxia.^[88]^ In diabetes, there an increased tendency for RBCs to aggregate or stick together, forming clusters. This hyperaggregability can obstruct blood flow in the microvasculature, leading to reduced blood perfusion and tissue ischemia. Additionally, RBCs may adhere more readily to the endothelial lining of blood vessels in diabetes, further impeding blood flow and contributing to microvascular complications.^[89]^ Diabetes causes endothelial dysfunction, characterized by impaired function of the cells lining the blood vessels (endothelial cells). This dysfunction contributes to alterations in vasodilation, increased vascular permeability, and a pro-inflammatory environment. These changes exacerbate microcirculatory disturbances, promoting microangiopathy.^[90]^ The combination of increased blood viscosity, altered RBC deformability, aggregation, adhesion, and endothelial dysfunction significantly contributes to micro-angiopathic complications commonly observed in diabetes. Management of these micro-angiopathic complications in diabetes involves comprehensive control of blood glucose levels, blood pressure, lipid levels, and lifestyle modifications. Additionally, treatments aimed at improving hemorheological parameters, such as medications to reduce blood viscosity or therapies targeting endothelial dysfunction, may be beneficial. Regular medical follow-ups and early intervention are crucial to prevent or delay the progression of these complications. Consulting healthcare professionals for personalized treatment plans and advice is essential for individuals with diabetes.

## 14. Vascular endothelial cell dysfunction in diabetes

Vascular endothelial cell dysfunction is a hallmark characteristic of diabetes, contributing significantly to the development and progression of various complications associated with the disease. Endothelial cells form a crucial inner lining of blood vessels and play a pivotal role in maintaining vascular health and function.^[91]^ Normally, endothelial cells produce NO, a potent vasodilator that helps regulate blood vessel tone. In diabetes, reduced bioavailability and impaired signaling of NO occur due to decreased activity of endothelial NO synthase and increased oxidative stress. This imbalance leads to impaired vasodilation and endothelial-dependent relaxation, contributing to hypertension and impaired blood flow regulation.^[92]^ Diabetes induces alterations in endothelial cell structure and function, leading to increased vascular permeability. This heightened permeability allows for the leakage of plasma proteins and other substances from the bloodstream into the surrounding tissues. In organs like the kidneys and eyes, increased vascular permeability contributes to diabetic nephropathy (kidney damage) and diabetic retinopathy (damage to the retina), respectively.^[93]^

Endothelial dysfunction in diabetes triggers an inflammatory response characterized by increased expression of adhesion molecules such as intercellular adhesion molecule 1 and vascular cell adhesion molecule 1. These molecules facilitate the attachment and migration of immune cells, promoting a chronic inflammatory state within blood vessels. The persistent inflammation contributes to atherosclerosis, the formation of plaques within arteries that can lead to cardiovascular complications like heart attacks and strokes.^[91]^ Altered endothelial function in diabetes results in an imbalance in the production of factors involved in blood clotting and fibrinolysis. There is an increased expression of procoagulant factors like tissue factor and reduced expression of anticoagulant factors like thrombomodulin and tissue plasminogen activator. This imbalance predisposes individuals with diabetes to a hypercoagulable state, potentially leading to thrombotic events.^[92]^ Diabetes induces the production of excessive ROS and reduces antioxidant defenses in endothelial cells. This oxidative stress damages cellular components, impairs endothelial cell function, and exacerbates the progression of vascular complications.^[94]^ Tight glycemic control is essential to mitigate the damaging effects of high glucose levels on endothelial cells. Healthy lifestyle choices including regular exercise, a balanced diet, smoking cessation, and weight management can positively impact endothelial health. Some medications like ACE inhibitors, angiotensin receptor blockers, statins, and drugs that target oxidative stress may help improve endothelial function and reduce the risk of vascular complications in diabetes. Regular monitoring, early detection, and prompt intervention are crucial to managing endothelial dysfunction in diabetes and reducing the risk of associated complications. Consulting healthcare professionals for tailored treatment plans and ongoing care is essential for individuals living with diabetes.

## 15. Conclusions

The intricate involvement of RBCs in diabetes mellitus and its complications underscores their significance as both biomarkers and mediators in the disease process. Throughout the diabetic milieu, RBCs undergo alterations in structure, function, and physiology, influencing disease progression and complications.

From a diagnostic perspective, HbA1c remains a cornerstone biomarker, reflecting long-term glycemic control. However, exploring beyond HbA1c reveals a spectrum of RBC-related biomarkers, such as oxidative stress markers and membrane alterations, offering comprehensive insights into diabetic pathophysiology.

Functionally, RBCs play a critical role as mediators in various aspects of diabetes complications, particularly microvascular dysfunction, oxidative stress, inflammatory responses, and tissue hypoxia. These alterations contribute significantly to the development and progression of diabetic retinopathy, nephropathy, neuropathy, and cardiovascular complications.

Understanding the role of RBCs in diabetes paves the way for therapeutic implications and future directions in managing the disease. Strategies targeting oxidative stress reduction, enhancing RBC functionality, personalized medicine, and innovative approaches may offer promising avenues for more effective interventions and tailored treatments.

In conclusion, the multifaceted role of RBCs in diabetes signifies their importance not only as diagnostic indicators but also as active participants in the disease process. Continued research into RBC-related biomarkers, advanced therapeutic approaches, and personalized interventions holds immense potential for improving diabetes management, mitigating complications, and ultimately enhancing the quality of life for individuals affected by diabetes mellitus.

## Author contributions

**Conceptualization:** Emmanuel Ifeanyi Obeagu.

**Methodology:** Emmanuel Ifeanyi Obeagu.

**Supervision:** Emmanuel Ifeanyi Obeagu.

**Validation:** Emmanuel Ifeanyi Obeagu.

**Visualization:** Emmanuel Ifeanyi Obeagu.

**Writing – original draft:** Emmanuel Ifeanyi Obeagu.

**Writing – review & editing:** Emmanuel Ifeanyi Obeagu.
